# C936T polymorphism of the *VEGF* gene in relation to the risk and the clinical and biological characteristics of sporadic colorectal adenocarcinoma

**DOI:** 10.1186/1756-0500-7-768

**Published:** 2014-10-31

**Authors:** Laura Credidio, Carmen Silvia Passos Lima, Raquel Leal, Maria Lourdes S de Ayrizono, João José Fagundes, Luis Alberto Magna, Claudio Saddy Rodrigues Coy

**Affiliations:** Department of Coloproctology, University of Campinas, Rua Carlos Chagas, 420, Cidade Universitária, Campinas, SP CEP 13083-878 Brazil; Department of Oncology, University of Campinas, Campinas, São Paulo Brazil; Department of Genetics, University of Campinas, Campinas, São Paulo Brazil

**Keywords:** Genetic polymorphism, Colorectal cancer, Vascular endothelial growth factor, Risk, Angiogenesis

## Abstract

**Background:**

One of the main glycoproteins responsible for angiogenesis is the vascular endothelial growth factor. It is believed that C936T polymorphism, located in the *VEGF* gene, is correlated with susceptibility towards development of sporadic colorectal adenocarcinoma. The aim of this study was to identify the frequencies of the genotypes of C936T polymorphism of the *VEGF* gene in patients with sporadic colorectal adenocarcinoma, in comparison with controls, and whether this correlates with the degree of tumor invasion, lymph node involvement and occurrence of metastases at the time of the diagnosis. The analysis was done on 261 patients with sporadic colorectal adenocarcinoma and 261 controls. The genotypes of C936T polymorphism were evaluated by means of the polymerase chain reaction and enzyme digestion, using peripheral blood samples.

**Results:**

The occurrences of genotype 936CC were similar in the two groups (80.5% versus 78.5%, p = 0.2288). In relation to tumor location, lymph node involvement, infiltration and tumor metastasis, no statistically significant results were obtained (p = 0.3116, p = 0.8485, p = 0.9408 and p = 0.2861, respectively).

**Conclusion:**

C936T polymorphism of the *VEGF* gene did not influence the occurrence of sporadic colorectal adenocarcinoma development and did not correlated with the degree of tumor invasion, lymph node involvement and occurrence of metastases.

## Background

Sporadic colorectal adenocarcinoma (SCA) is the third most frequent type of cancer among men and the second among women in the southern and southeastern regions of Brazil [1]. According to the Heath Department of Campinas, this type of cancer is the second biggest cause of death among women [2]. One of the main glycoproteins responsible for angiogenesis, with important action relating to cell proliferation survival and migration, along with mediation of vascular permeability, is the vascular endothelial growth factor (VEGF) and its receptors [3–5]. Increased expression of VEGF has been correlated with worse prognosis in colorectal cancer cases [6–8].

C936T polymorphism (rs3025039) is located in the *VEGF-A* gene, and consists of replacement of a single nucleotide (SNP) in exon 8 in the non-translated 3’ region (3’UTR) [9], with replacement of the cytosine base (C) by thymine (T) [10–13], thus resulting in loss of the region that transcribes the factor AP-4 (activated protein 4). Analyses on the complementary DNA product (cDNA) have suggested that AP-4 codes for the protein HLH, and that this protein recognizes specific sites in the CAGCTG DNA sequence [14].

In the presence of the wild genotype, the glycoprotein level in plasma increases, thus stimulating formation of new vessels [15–19]. According to RENNER et al. [20], individuals with the genotype variants CT and TT have low serum levels of VEGF, thus diminishing angiogenesis and the risk of acquiring cancer. This hypothesis has been corroborated by other authors, who found that the T allele had a protective effect regarding the risk of developing malignant neoplasm or even a less aggressive tumor [12, 15, 21, 22]. On the other hand, Hofmann et al. [21] and Dassaoulas et al. [22] did not identify any association between SCA and C936T polymorphism of the *VEGF* gene. A meta-analysis conducted by Xu et al. [23] containing 13,293 cancer cases and 12,308 controls did not show any association between SCA and C936T polymorphism of the *VEGF* gene. However, other authors have shown that the T allele was associated with higher risk of SCA and other type of malignant neoplasm [18], colorectal adenoma [24], gastric cancer [19] and oral cancer [25]. These authors believed that the heterozygote variant genotype CT was responsible for the increased occurrence of neoplasm, but also stated that the results could vary between different ethnic groups, with different expressions in different populations.

Some authors have compared the histopathological characteristics of SCA with this polymorphism, but the results have been divergent. Neither Hofmann nor Dassoulas found that *VEGF* C936T polymorphism had association with size, tumor differentiation, tumor stage and presence of lymph node metastasis or age group. Chae et al. [26] obtained similar results, but Lurje et al. [27] demonstrated that the wild genotype CC was related to recurrence in stage III of SCA.

The aim of this study was to investigate whether *VEGF* C936T polymorphism influenced the risk of development of SCA, and whether, in individuals with this neoplasm, it might correlate with the degree of tumor invasion, lymph node involvement and occurrence of metastases at the time of the diagnosis. The Ethics Committee of the School of Medical Sciences, UNICAMP, approved this study under opinion report no. 759/2007.

## Methods

Assessments were made on 261 patients with SCA, both genders, of mean age 65.2 years (range: 32–97), and 261 controls comprising blood donors of both genders and mean age 52.9 years (range: 25–60). The groups were matched according to sex and racial group. Consent for participation in the study was obtained from all participants. The characteristics analyzed were tumor location, lymph node involvement, occurrence of metastases and tumor stage. The tumor was established in accordance with the criteria of the AJCC Cancer Staging Manual [28]. With regard to tumor location, tumors were described as right-colon tumors if they were in the cecum, ascending colon or transverse colon; left-colon tumors if they were in the descending colon or sigmoid; or rectal tumors if they were located up to 18 cm from the anal margin. In relation to tumor infiltration, the patients were divided into two groups: Tis, T1 and T2 lesions; or T3 and T4 lesions. With regard to lymph node involvement, occurrence or non-occurrence of metastasis was taken into account, independent of the number of lymph nodes involved. In terms of the TNM classification, the patients were grouped into states I and II versus III and IV.

C936T polymorphism was evaluated in peripheral blood samples. The polymorphism analysis was done in accordance with the methods described by Renner *et al.* [21], using the polymerase chain reaction, followed by enzyme digestion using the endonuclease restriction enzyme *Nla*III. The forward primers used was 5’- AAG GAA GAG ACT CTG CGC AGA GC - 3’ and the reverse primer was 5’ - TAA ATG TAT GTA TGT GGG TGG GTG TGT CTA CAG G - 3’.

A test to verify Hardy-Weinberg equilibrium was performed with the aim of finding out whether there was any preferential distribution of any of the genotypes evaluated in the patient and control groups of this study. The statistical significance of the differences between the groups was calculated by means of the Fisher exact test and chi-square test, using the “diagnose parameters α” software (developed by one of the collaborators), Epi Info version 6.04d (CDC, Atlanta, Georgia, USA) and Excel 2010 (Microsoft). The statistical significance level was set at p < 0.05.

## Results

The sample size calculation, according to the frequencies of polymorphisms was 237.72. The Hardy-Weinberg test was applied to both controls and patients. Both were in equilibrium (*X*^2^ = 0.019, p = 0.89; and *X*^2^ = 0.587, p = 0.4436, respectively), for the locus of *VEGF* C936T polymorphism. Similar frequencies were seen between patients and controls, with a relative risk (OR) of 1.47 (95% CI: 0.80-2.70), adjusted according to age and frequency between the genotypes, without any statistically significant result (p = 0.737) (Table 1).

**Table 1:** **VEGF genotypes in sporadic colorectal cancer patients and controls**

	Patients: number and%	Controls: number and%	OR	P
**CC**	210 (50.6)	205 (49.4)	1.00 (reference)	0.7370
**CT**	48 (47.1)	54 (52.9)	1.14 (0.75 to 1.72)	
**TT**	3 (60)	2 (40)	0.68 (0.11 to 4.10)	

To analyze the polymorphism, 261 patients with sporadic colorectal adenocarcinoma and 261 controls were evaluated. With regard to tumor infiltration and lymph node involvement, 93 patients with rectal neoplasm and neoadjuvant therapy were excluded. One case was not classified in relation to tumor location because of lack of data in the medical records, and 19 did not undergo surgical treatment and did not present a TNM classification. Table 2 shows the number of patients analyzed in relation to the characteristics analyzed.

**Table 2:** **Patients analyzed**

	Number of patients	Number of controls	Patients excluded
**VEGF polymorphism**	261	261	-
**Metastasis**	261	-	-
**Tumor location**	260	-	1
**Survival**	258	-	3
**Infiltration (T)**	165	-	96
**Lymph node (N)**	165	-	96

No significant differences were identified in relation to tumor invasion (p = 0.9408; OR = 1.0968, 95% CI: 0.4479-2.6854), lymph node involvement (p = 0.8485; OR = 0.9275, 95% CI: 0.4286-2.0072), occurrence of metastases (p = 0.2861; OR = 1.0361, 95% CI: 0.5010-30.9979), tumor location (p = 0.3116, OR = 1.0361, 95% CI: 0.4798-2.2375) or TNM (p = 0.6045; OR = 1.0550, 95% CI: 0.5466-2.0363) (Table 3).

**Table 3:** **VEGF genotype in sporadic colorectal cancer patients stratified according to clinical characteristics**

Clinical characteristics	Number of patients (%)	C936T VEGF polymorphism		
		CC (%)	CT + TT (%)	P value
**Mean age at diagnosis**	261	64.9	65.8	
**Ethnic origin**	261			
Caucasian	219 (84,9)	173 (66,3)	46 (17,6)	0.4693
African-American	42 (16,1)	33 (12,6)	9 (3,4)	
**Metastasis**	261			0.7843
No	211 (80,8)	166 (63,6)	45 (17,2)	
Yes	50 (19,2)	40 (15,3)	10 (3,8)	
**Survival**				0.2619
≥ 5 years	147	112 (76,2)	35 (23,8)	
**Regional lymph node**	165			
N+	80 (48,5)	64 (38,8)	16 (9,7)	0.8485
N0	85 (51,5)	69 (41,8)	16 (9,7)	
**Infiltration**	165			
Tis-T2	39 (23,6)	31 (18,8)	8 (4,8)	0.9408
T3-T4	126 (76,4)	102 (61,8)	24 (14,5)	
**Tumor location**	260			
Rectum	137 (52,7)	106 (41,1)	29 (11,2)	0.3116
Left Colon	79 (30,4)	60 (23,3)	19 (7,4)	
Right Colon	44 (16,9)	39 (15,1)	5 (1,9)	
**TNM**	226			0.6045
I + II	139 (61,5)	109 (48,2)	30 (13,3)	
III + IV	87 (38,5)	69 (30,5)	18 (8)	

## Discussion

Angiogenesis performs an essential role in tumor growth and invasion, and in occurrence of metastases, and it may be stimulated through production of factors generated by neoplastic cells [29, 30]. Among the factors that regulate angiogenesis, VEGF is the best known and, given its capacity to stimulate vascular proliferation, its expression is thought to have the capacity to modify the biological behavior of solid tumors, including SCA. However, the human *VEGF* gene is highly polymorphic, thus enabling wide variation in its expression between individuals.

It is now known that VEGF is not limited to angiogenesis and vascular permeability, it can also be an autocrine and damage the functions of immune cells found in the tumor microenvironment and therefore affecting the mass response to tumors. The increase in the synthesis of VEGF can be detected plasma in advanced tumors. The VEGF receptor (VEGFR1) promotes the migration and invasion of colorectal tumor cells besides maintaining their survival [31]. In the presence of wild genotype (CC), glycoprotein level in plasma increases, stimulating the development of new vessels [15, 16, 18, 19, 32].

It is believed that *VEGF* C936T polymorphism interferes with production of the protein VEGF [20]. The homozygote variant genotype TT has been associated with lower plasma levels of this protein [27]. Thus, the TT genotype would diminish angiogenesis and, in theory, would give rise to less tumor development. It is known that higher preoperative serum levels of VEGF in individuals with SCA has been associated with lower survival [33].

However, studies on this polymorphism in different solid tumors have presented different results in relation to the risk of cancer or prognostic factors [9, 13, 34]. Oliveira *et al.* [34] demonstrated that the wild genotype (CC) is a determinant for greater aggressiveness of sporadic breast tumors.

In gastric cancer cases, Tzanakis *et al.* [19] found that a strong association between the C936T gene and the TT genotype was related to greater tumor size. However, the results from the present study, along with the results from Hofmann and Dassoulas, do not support the existence of associations with tumor size, histological grade, tumor stage, lymph node metastases or age at the time of diagnosis, in SCA cases. On the other hand, Chae [26] showed that the T allele was related to the advanced stage of SCA (metastasis).

These data suggest that the contribution of each genotype to the tumor characteristics may be closely related to the type of organ that is bearing the malignant growth.

The present study suggests that the genotypes studied did not increase the risk of SCA (CT vs. CC = OR 1.47, 95% CI: 0.80 to 2.70; and TT vs. CT = OR 0.34, 95% CI: 0.03 to 3.63). These findings were corroborated by Hofmann [22], Dassaoulas [23], Xu [24] and coworkers, obtaining the same results in relation to C936T polymorphism. Divergent findings were obtained by Eroglu and Wu [18, 35], in a study that showed that this polymorphism increased the patient’s predisposition towards developing a malignant tumor, even though the frequencies of the variants in their study were similar to those in other studies [12, 36].

These data can be explained by the action of a variety of factors, such as the variation in the locus, action by several other genes, environmental characteristics and ethnicity, which therefore provides justification for studying this in different populations. Hefler *et al.* [37] reported that simultaneous presence of three homozygote genotypes (2578/CC, 1154/GG and 634/CC) increased the serum levels of VEGF and was correlated with shorter survival, among 563 patients with ovarian cancer. Thus, in addition to the sequential variation in the *VEGF* gene that may alter its production or activity, other angiogenic factors present in the circulation could, through interacting with *VEGF*, influence its role in the pathogenesis of cancers.

The analysis on polymorphism in relation to location, tumor infiltration, lymph node involvement, occurrence of metastases and staging did not show any significant differences. These findings differed from those of Bae *et al.* [36], who observed that occurrences of the T allele increased the risk in men under the age of 55 years and in all women, and that this was associated with greater occurrence of adenocarcinoma in the right colon. These results can be explained in terms of ethnic differences.

In the present study, C936T polymorphism did not change the risk of developing SCA. Our results are concordant with those of Hofmann *et al.* [21], Dassoulas *et al*. [22] and Wu *et al.* [35], who suggested that the C2578A, G634C and C936T polymorphisms were not general risk factors for SCA. Analysis on C936T polymorphism of the *VEGF* gene, as done in the present study, has also been conducted on cases of breast, lung and stomach cancer and on melanoma [9, 19, 38]. C936T polymorphism of the *VEGF* gene was found not to significantly influence the susceptibility to breast cancer in the study by Jin *et al.* [39]. On the other hand, individuals with the T allele in cases of VEGF 936 presented diminished risk of breast and lung cancer, according to other researchers [12, 13]. These results are not unexpected, since VEGF functions as a fundamental mediator of angiogenesis, thereby influencing biological and phenotypic characteristics (tumor size, histological grade, disease stage and metastatic potential), rather than defining the susceptibility to cancer development.

## Conclusion

Polymorphism of the *VEGF* C936T gene did not influence the occurrence of SCA development and was not correlated with the degree of tumor invasion, lymph node involvement or occurrence of metastases.
